# Monitoring dendritic cell and cytokine biomarkers during remission prior to relapse in patients with FLT3-ITD acute myeloid leukemia

**DOI:** 10.1007/s00277-013-1744-y

**Published:** 2013-04-25

**Authors:** Mareike Rickmann, Laura Macke, Bala Sai Sundarasetty, Kathrin Stamer, Constanca Figueiredo, Rainer Blasczyk, Michael Heuser, Juergen Krauter, Arnold Ganser, Renata Stripecke

**Affiliations:** 1 Hematology, Hemostasis, Oncology and Stem Cell Transplantation, Hannover Medical School (MHH), OE 6860, Carl-Neuberg Str. 1, 30625 Hannover, Germany; 2 Department of Transfusion Medicine, MHH, Hannover, Germany

**Keywords:** Leukemia, Dendritic cell, Immune monitoring, Myeloid-derived suppressor cells, Risk of relapse, Inflammatory cytokines

## Abstract

Relapse occurs frequently after treatment of acute myeloid leukemia (AML) patients with the FMS-like tyrosine kinase 3-internal tandem duplication (ITD) mutation. The availability of immunologic biomarkers to predict patients at high risk could allow clinicians to accelerate alternative treatments such as stem cell transplantation, immunotherapy, or novel drugs. We have previously reported that first diagnostic (FD) ITD^+^ AML showed immunophenotypic and functional characteristics of arrested dendritic cell (DC) precursors. In this study, we show that the high frequency of precursor DCs in 16 FD ITD^+^ AML samples (Lin^−^/HLA-DR^+^/CD11c^+^/CD123^+^) was associated with a lack of terminal DCs (myeloid DCs: BDCA-1^+^ or BDCA-3^+^; plasmacytoid DC: BDCA-2^+^). We further evaluated prospectively the peripheral blood complete remission (CR) samples obtained from 11 ITD^+^ AML patients after chemotherapy regarding the frequency of DCs and their pattern of cytokine production. Whereas the aberrant frequencies of precursor and terminal plasmacytoid DCs resolved during remission, the myeloid DC compartment did not fully recover. For an available cohort of patients (*n* = 4) who could be monitored over a period of >15 months after FD, we identified IL-10, TNF-α, IL-6, and IL-1β as cytokines produced by the CR samples at high levels a few months prior to relapse. Cell-free supernatant of an FD ITD^+^ AML sample stimulated monocytes obtained from two healthy donors to secrete IL-10, TNF-α, IL-6, and IL-1β. Thus, we hypothesize that ITD^+^ AML minimal residual disease can act directly as dysfunctional antigen-presenting cells or indirectly by production of factors that convert monocytes into myeloid-derived suppressor cells secreting cytokines that promote immune evasion. Monitoring these immunologic biomarkers could improve prediction of relapse.

**Electronic supplementary material:**

The online version of this article (doi:10.1007/s00277-013-1744-y) contains supplementary material, which is available to authorized users.

## Introduction

Acute myeloid leukemia (AML) is a very heterogeneous disease and risk stratification has recently been improved by detection of genetic mutations in samples collected at first diagnosis (FD) and complete remission (CR) [1]. One of the most common mutations in AML occurs in the open reading frame of FMS-like tyrosine kinase 3 (FLT3), which is the receptor for FLT3-ligand, a cytokine with pivotal function in dendritic cell (DC) differentiation and in stem cell renewal [2, 3]. The mutations in the FLT3 receptor occur by point mutations in the kinase domain or by internal tandem duplications (ITD) of the juxtamembrane or tyrosine kinase domain. These mutations afflict approximately 30 % of the newly diagnosed AML cases corresponding to a group of patients at high risk [4, 5]. Once FLT3-ITD is identified, mutations in other loci can be used to aid in the outcome prediction (such as NPM1, MLL, N-RAS, and WT1) [5–7]. Novel sensitive approaches to detect leukemia relapse through next-generation sequencing of mutated hotspots such as FLT3-ITD and NPM1 are currently in development [8].

Clinical evidence indicates that allogeneic stem cell transplantation (SCT) of ITD^+^ AML patients results in the best survival outcome and is the routine clinical practice for eligible patients [9]. The mechanistic effects of allo-SCT are believed to be largely immunological, due to the capacity of T cells from the donor to mount a curative and long-term graft-versus-leukemia effect against minimal residual disease (MRD) [10]. Unfortunately, older patients and patients without suitable donors are not eligible for allo-SCT and conventional chemotherapy is the standard of care. For these patients, novel therapies in clinical development such as immunotherapy or novel drugs are certainly warranted. In addition, novel diagnostic approaches such as immune monitoring would potentially impact in the prediction of MRD immune evasion and relapse.

Dendritic cells play a key role in immune regulation and function [11, 12]. The DC compartment in cancer patients is often deregulated, resulting in the accumulation of immature and/or dysfunctional DCs [13]. We have recently shown that FD samples of adult ITD^+^ AML patients contain an aberrant accumulation of cells with immunophenotypic and functional characteristics of arrested DCs [14]. Thus, we hypothesize that residual leukemic ITD^+^ DCs could potentially affect the differentiation of normal DCs of patients during disease recovery through paracrine mechanisms. In that regard, we evaluated if cytokines (IL-10, TNF-α, IL-6, and IL-1β) proposed by several groups as indicators of a broad range of immunopathological conditions affecting cancer [15–20] could also serve as potential biomarkers to predict ITD^+^ leukemia relapse.

In this study, we prospectively collected samples from 11 ITD^+^ AML patients treated with standard chemotherapy. Cohorts of nonresponders, patients who entered remission and then relapsed or patients with relapse-free survival for >15 months, were identified. Peripheral blood samples of ITD^−^ AML patients and healthy donors (HD) were used as control groups. Kinetic analyses of four ITD^+^ AML patients who entered CR but then relapsed between 16 and 24 months after FD showed a consistent pattern of arrested terminal differentiation of myeloid DCs and upregulation of IL-10 (an anti-inflammatory cytokine), TNF-α, IL-6, and IL-1β (pro-inflammatory cytokines) months prior to leukemia relapse. We provided experimental evidence that soluble factors produced by ITD^+^ AML stimulated monocytes from healthy donors to produce IL-10, TNF-α, IL-6, and IL-1β.

## Materials and methods

### Patient samples

This study was performed in accordance with the declaration of Helsinki and was approved by the local ethics committee of the Hannover Medical School. Peripheral blood samples from adult (18–83 years) AML patients were collected after written informed consent. Twenty-six ITD^+^ and 28 ITD^−^ patients, for whom FD samples were available, were included in this study (Suppl. Table 1). Peripheral blood mononuclear cells (PBMCs) were collected from 26 ITD^+^ patients for up to 24 months after FD and they were grouped according to the clinical outcome (Suppl. Fig. 1b and Table 1): nonresponders (NR, no CR after induction therapy, i.e., >5 % blasts in the BM) and CR. CR patients were split between those who eventually relapsed >15 months after FD (CR/REL) and those patients with disease-free survival (CR/DFS, no relapse up to 2 years after FD).

**Table 1:** Patient characteristics of ITD^+^ patient groups NR, CR/REL, and CR/DFS

ID	Sex	FAB	WBC	Age	Karyotype	Mutations			REL (month)
						FLT3 ITD	NPM1	WT1	
NR									
#1	F	M4	1.4	57	46, XX	+	−	+	−
#2	M	M5	n/a	45	Complex	+	−	+	−
#3	M	M5	119.2	57	n/a	+	−	−	−
#4	M	sAML	259	67	n/a	+	−	n/a	−
#5	M	n/a	97	83	n/a	+	+	+	−
CR/ REL									
#6^a^	M	M4	110.1	40	46, XY	+	+	+	18
#7 ^a^	F	M4	98.1	53	46, XX	+	+	+	16
#8 ^a^	F	M5	66.7	48	Complex	+	−	+	20
#9 ^a^	F	M2	69.9	52	46, XX	+	+	+	24
#10	M	M5a	56.2	44	46, XY	+	+	n/a	10
#11	M	M4	9	24	46, XY	+	−	+	3
#12	F	M4	0.4	78	Complex	+	−	+	3
#13	F	M5a	107	53	46, XX	+	+	n/a	8
CR/ DFS									
#14	F	M4	6.3	64	46, XX	+	+	+	No REL
#15	F	M1	n/a	66	46, XX	+	+	+	No REL
#16	M	M4	3.1	77	46, XY	+	+	+	No REL
#17	M	n/a	n/a	32	n/a	+	−	n/a	No REL
#18	F	M2	243	70	46, XX	+	+	n/a	No REL
#19	M	n/a	n/a	40	n/a	+	+	n/a	No REL
#20	F	M4	45.2	63	46, XX	+	+	+	No REL
#21	F	M1	3.6	50	46, XX	+	+	+	No REL
#22	M	n/a	12	40	46, XY	+	−	+	No REL
#23	F	M3	n/a	65	APL	+	−	n/a	No REL
#24	F	M5	n/a	61	46, XX	+	−	n/a	No REL
#25	M	M6	5.9	18	46, XY	+	+	n/a	No REL
#26	M	M5b	n/a	67	46, XY	+	+	n/a	No REL

*NR* nonresponders, *CR/REL* complete remission followed by relapse (within 2 years from first diagnosis), *CR/DFS* complete remission and disease-free survival (for >2 years from first diagnosis), *FAB* French American British Classification, *WBC* white blood cells ×1,000/μl, *NPM1 mutation* exon 12 nucleophosmin mutation, *WT1* Wilm's tumor 1 gene mutation, *REL* relapse, *n/a* not available

^a^Patients analyzed for kinetics of late leukemia relapse and correlation with DC and cytokine profile

### Cytogenetics, FLT3-ITD, and WT1 analyses

Cytogenetic and molecular genetic studies (Table 1 and Suppl. Table 1) were performed by the German–Austrian Acute Myeloid Leukemia Study Group at Hannover Medical School or at the University of Ulm. Blood diagnostic samples were analyzed for the presence of the ITD mutations in the FLT3 gene by polymerase chain reaction as described previously [21]. The upregulation of WT1 mRNA levels was used as a putative approach to predict relapse as previously described [22]. RNA was extracted from approximately 5 × 10^6^ PBMCs using the Qiagen RNeasy kit according to the protocol of the manufacturer (Qiagen, Hilden, Germany). Real-time quantitative polymerase chain reaction (RQ-PCR) was performed with extracted RNA using the WT1 Profile*Quant*®-Kit (ELN) (Ref: PQPP-02-CE) from Ipsogen (Luminy Biotech Enterprises, Marseille, France). The RQ-PCR consists of two holding stages of 1 cycle each at 50 °C for 2 min followed by 95 °C for 10 min and a cycling stage at 95 °C for 15 s and 60 °C for 1 min (50 cycles) in a StepOnePlus Real-Time PCR system (Applied Biosystems, Darmstadt, Germany). Standard curves and fluorescence calculation were performed using the StepOnePlus Real-Time PCR system and Microsoft Excel (Microsoft, Germany).

### Immunophenotypic analyses of precursor and terminal DCs

PBMCs obtained from patients and healthy volunteers were isolated by standard density gradient centrifugation using Ficoll (Biocoll separating solution, Greiner, Bio-One, Germany) separation and cryopreserved in 90 % FBS and 10 % DMSO. Progenitor DCs were identified using a commercially available kit (“Peripheral Blood Dendritic Cell Detection,” Becton Dickinson BD, San Jose, CA, USA). The protocol is based on a four-color staining. For the detection of myeloid and plasmacytoid progenitor DCs, we used lineage cocktail 1 (FITC) containing monoclonal antibodies (mABs) against CD3, CD14, CD16, CD19, CD20, and CD56 as a negative selection, a mAB against HLA-DR (PerCp, clone L243), a mAB against the CD11c myeloid DC marker (APC, clone S-HCL-3), and a mAB against the CD123 plasmacytoid DC marker (PE, clone 95F). Fifty thousand viable cells gated on the forward scatter (FSC)/side scatter (SSC) were negatively selected using the lineage markers. The resulting Lin^−^ population was analyzed for HLA-DR/CD11c (“precursor myeloid dendritic cells (mDCs)”) or HLA-DR/CD123 (“precursor plasmacytoid dendritic cells (pDCs)”). Terminal DCs were identified using a commercially available kit (“Blood Dendritic Cell Enumeration,” Miltenyi Biotec, Bergisch-Gladbach, Germany). The protocol is based on a four-color staining: for mDC1-, a mAB against BDCA-1 (CD1c, PE); for pDC-, a mAB against BDCA-2 (CD303, FITC); and for mDC2 detection, a mAB against BDCA-3 (CD141, APC). We used a photoaffinity fluorescent “dead cell discriminator” (PE-Cy5) and mABs against CD19 and CD14 to (PE-Cy5) to exclude B cells, monocytes, granulocytes, and dead cells. Fifty thousand viable cells gated on the FSC/SSC scatter and excluding dead cells and CD19^+^/CD14^+^ cells were analyzed for expression of BDCA-1 (mDC1), BDCA-2 (pDC), and BDCA-3 (mDC2). Stained cells were analyzed with a FACSCalibur using CellQuest software (BD, San Jose, CA, USA).

### Analyses of cytokine secretion

Cell-free supernatants of PBMC samples obtained from leukemia patients were obtained by seeding of 1 × 10^6^ viable cells in 1 ml of X-vivo medium (Lonza, Belgium) in 12-well plates (TPP, Switzerland), incubated for 24 h at 37 °C and 5 % CO_2_, collected, centrifuged to remove cells and debris, and cryopreserved at −80 °C. Luminex® analyses were performed with a Cytokine Human 14-Plex Panel (Millipore, Schwalbach, Germany) for analyses of IL-10, TNF-α, IL-6, and IL-1β. As controls, supernatants obtained from PB samples obtained from four healthy donors were included in the analyses. Cytokine standards supplied by the manufacturer were run on each plate together with the test samples. Samples were treated accordingly to manufacturer's instructions. For cytokine detection, data were acquired on a Luminex-200 System and analyzed with the Xponent software v.3.0 (Invitrogen).

### Treatment of CD14^+^ monocytes with AML supernatant

The cell culture supernatant collected from one of the FD samples in the study (CR/REL patient #6) as described above was filtered with 0.2 μM and diluted with X-vivo medium at dilutions of 1:3 and 1:9. CD14^+^ monocytes were isolated from the leukapheresis of healthy donors using CD14 microbeads (Miltenyi, Germany). The isolated CD14^+^ monocytes were seeded at a density of 5 × 10^6^ cells per well and were cultured in X-vivo medium or with diluted 2-ml AML supernatants in a six-well plate. The plates were incubated at 37 °C for 3 days. At the end of the incubation period, the cell viability and monocyte purity were assessed by flow cytometric analyses of 7AAD and CD14. The cell supernatants were collected and the cytokine profile was analyzed by Luminex® as described above.

## Results

### FD samples of ITD^+^ AML patients showed a more pronounced alteration of the dendritic cell pattern in comparison to FD samples of ITD^−^ AML patients

**Fig. 1:**
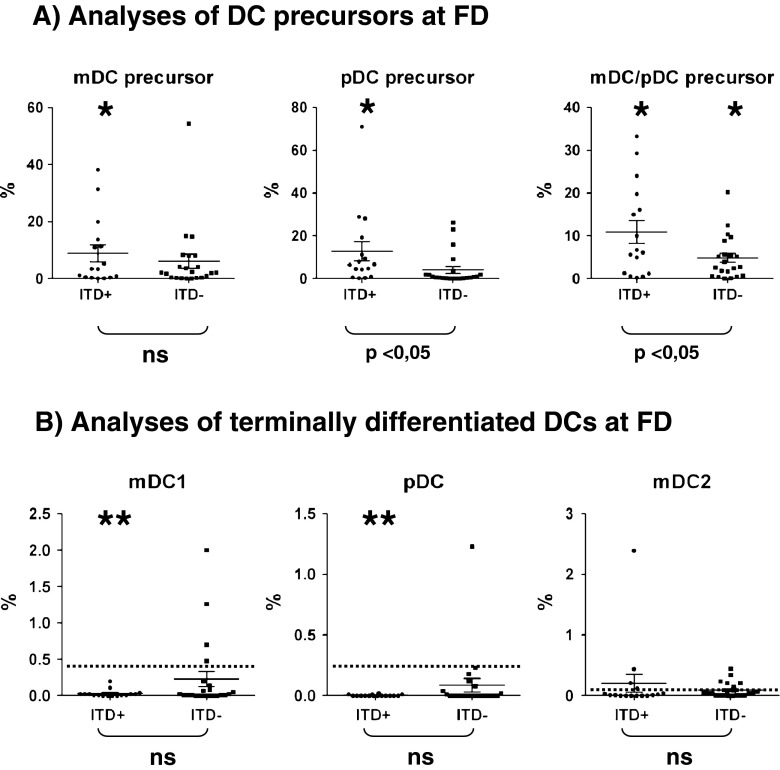
FD samples of ITD^+^ AML patients (*n* = 16) showed a more pronounced alteration of the dendritic cell pattern in comparison with the FD samples of ITD^−^ AML patients (*n* = 22). **a** Frequencies of precursor DCs. **b** Frequencies of terminal DCs. *Single asterisk:* significantly higher than HD (*p* < 0.05). *Double asterisk:* significantly lower than HD (*p* < 0.05). [The *dotted line* is showing the average values for HD]

We had previously reported that FD samples of ITD^+^ patients presented with an accumulation of Lin^−^/HLA-DR^+^ cells that co-expressed the DC markers CD11c and/or CD123. These leukemic DCs contained ITD mutations and could be driven to differentiate into mature mDCs or pDCs in vitro upon culture with cytokines, but they were not fully capable of secreting several cytokines [14]. We therefore deduced that CD11c^+^, CD123^+^, and CD11c^+^/CD123^+^ leukemic DCs were arrested in the dendritic cell differentiation pathway and that CD11c^+^ and CD123^+^ should be rather considered as DC precursor markers. In order to assess the frequencies of mature DCs in leukemia samples, we therefore used a second immunophenotypic panel. Here, terminal DCs were defined as mDC1 (BDCA-1^+^), pDC (BDCA-2^+^), or mDC2 (BDCA-3^+^). Validation of the immunophenotypic analyses was performed with cryopreserved/thawed PBMC obtained from nine healthy control donors. For both types of immunophenotypic analyses, we observed a higher frequency of mDCs (approximately 0.4 %) compared to pDCs (approximately 0.2 %) (Suppl. Fig. 1a). The frequencies of mixed lineage mDC/pDCs (CD123^+^/CD11c^+^) and of mDC2 were quite low (below 0.1 %). Using the same staining procedures, we examined FD-cryopreserved samples of ITD^+^ and ITD^−^ patients who were diagnosed and treated at the Hannover Medical School. We compared the FD samples of 16 ITD^+^ and 22 ITD^−^ patients, who eventually entered CR. The cohorts were reasonably similar in terms of gender distribution, age, WBC counts, NPM1 mutation frequency, FAB types, and cytogenetics (Suppl. Table 1). In contrast to the homogeneous frequencies of DCs in PBMC samples obtained from HD (0−0.83 %), FD samples from ITD^+^ and ITD^−^ patients showed a high variability in the frequencies of precursor DCs, ranging from 0 to 71 % (Fig. 1a and Suppl. Figs. 1a and 2a). We observed the previously reported accumulation of mDC/pDC precursors (CD123^+^/CD11c^+^) in FD obtained from ITD^+^ patients (Fig. 1a and Suppl. Fig. 2a). The frequencies of mDC/pDC precursors were significantly increased in both ITD^+^ (*p* = 0.0065) and ITD^−^ (*p* = 0.0077) FD patient samples compared to HD PBMCs. Notably, the accumulation of mDC/pDC precursors was significantly higher in the FD of ITD^+^ patients compared to the FD of ITD^−^ patients (mDC: *p* = 0.046; pDC: *p* = 0.049). The same samples demonstrated almost complete lack of terminal mDC1s (BDCA-1^+^) and pDCs (BDCA-2^+^) (Fig. 1b and Suppl. Fig. 2b). Of note, significantly lower frequencies of terminal mDC1 (*p* < 0.0001) and pDC (*p* < 0.0001) were found in FD samples of ITD^+^ patients compared to HD, whereas the frequencies of terminal DCs in FD samples of ITD^−^ were not significantly affected (Fig. 1b). Thus, these results fully corroborated with our previous findings using another AML cohort, showing that the ITD mutation in AML is correlated with the accumulation of mDC/pDC precursors. Furthermore, these leukemic ITD^+^ DC precursors did not display the terminally differentiated DC immunophenotype. These findings combined underscore the notation that ITD^+^ AML corresponds to an arrested DC lineage malignancy.

### FD samples obtained from ITD^+^ NR patients secreted higher levels of IL-10 and stress cytokines in vitro in comparison to CR patients

**Fig. 2:**
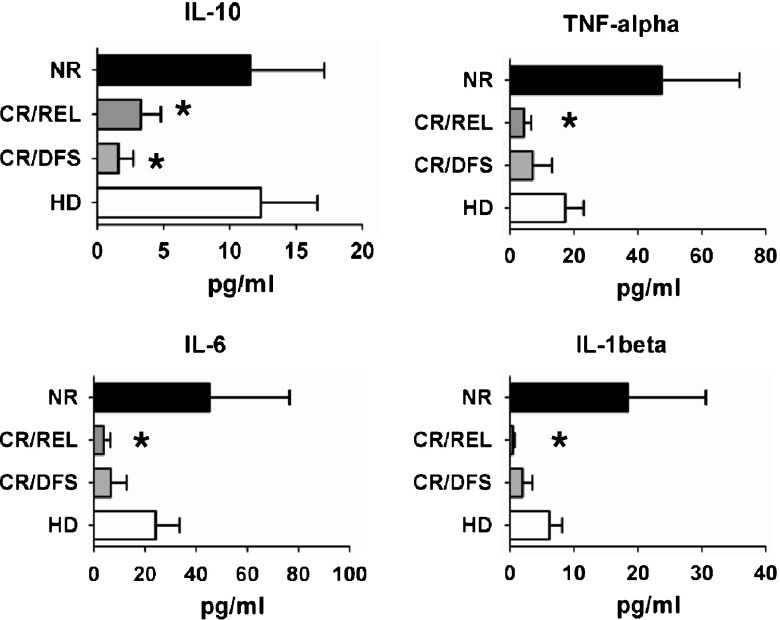
FD samples obtained from NR ITD^+^ AML patients (*n* = 4) secreted higher levels of stress cytokines in vitro in comparison with patients who entered CR (*n* = 7). *NR* nonresponders (*n* = 4), *CR/REL* complete remission followed by relapse (<2 years; *n* = 4), *CR/DFS* complete remission and disease-free survival (>2 years) (*n* = 3), *HD* healthy donors (*n* = 4). *Single asterisk:* significantly lower than HD *p* < 0.05. [Detection level for cytokines is 3.2 pg/ml]

IL-10 is produced mostly by macrophages and myeloid DCs and is generally regarded as an anti-inflammatory cytokine exerting several effects in immunomodulation of the cancer microenvironment and cancer progression [17]. Direct inhibition of myeloid DC differentiation mediated through IL-10 and IL-6 was proposed to induce a potentially immunocompromising microenvironment in cancer patients [23]. IL-6, TNF-α, and IL-1β are predominantly secreted by activated monocytes and macrophages and play a central role in innate inflammatory events. Remarkably, the neuroendocrine stress system in human beings is also triggered via soluble factors such as IL-6, TNF-α, and IL-1β, heretofore named “stress cytokines” [17, 24]. Therefore, we proposed to evaluate whether FD containing ITD^+^ leukemic cells would show particular patterns of expression of IL-10 and/or stress cytokines. FD samples obtained from NR patients showed higher levels of production of all stress cytokines compared with PBMC of HD, whereas IL-10 levels were similar (Fig. 2). On the other hand, FD samples obtained from ITD^+^ CR patients (CR/REL and CR/DFS) showed lower levels of secreted IL-10, TNF-α, IL-6, and IL-1β than PBMCs of HD controls (Fig. 2). Thus, compared with the DC immunophenotypic analyses of ITD^+^ AML showing a more uniform pattern, the cytokine secretion pattern was more heterogeneous. These findings suggested that either the ITD^+^ leukemic cells from NR patients secreted cytokines in a different pattern than cells obtained from CR/REL and CR/DFS, or that bystander cells contained in FD samples of NR patients could be responsible for the secretion of the cytokines.

### CR samples obtained from ITD^+^ AML patients showed lower recovery of myeloid DCs compared with samples obtained from ITD^-^AML patients

**Fig. 3:**
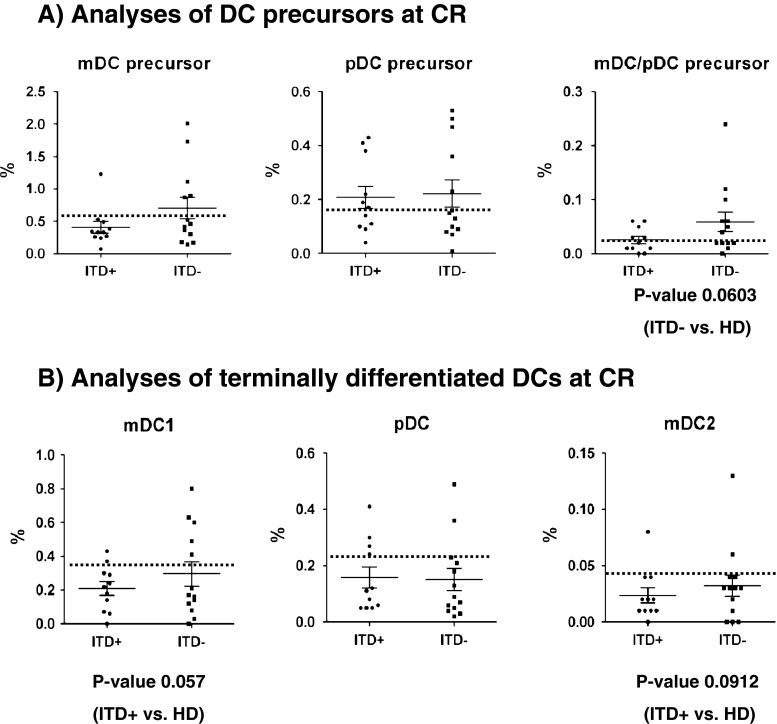
CR samples obtained from ITD^+^ AML patients (*n* = 11) showed suboptimal recovery of myeloid DCs compared with samples obtained from ITD^−^ AML patients (*n* = 13). **a** Normalization in the frequencies of precursor DCs. **b** Trend for lower frequencies of terminal mDCs. [The *dotted line* is showing the average values for HD]

Low DC frequencies in leukemia patients after initial treatment and/or HSCT have been associated with relapse and a poor outcome [25, 26]. Therefore, we compared the frequencies of precursor and terminal DCs in samples obtained from ITD^+^ (*n* = 11) and ITD^−^ (*n* = 13) patients in CR (Suppl. Fig. 1b and Table 1). During CR, the mDC/pDC precursor population disappeared in both the ITD^+^ and ITD^−^ cohorts (Fig. 3a). ITD^+^ CR patients showed a trend towards lower frequencies of terminal myeloid DCs (mDC1, *p* = 0.057; mDC2, *p* = 0.0912) in comparison to HD (Fig. 3b). These findings suggested that the aberrant DC patterns observed at ITD^+^ AML diagnosis tended to normalize during CR, but did not return to the normal situation observed in healthy individuals. Thus, despite of this modest alteration in DC frequency, we could not identify a major aberration in CR samples of ITD^+^ AML patients as a whole.

### Kinetic analyses of CR samples of ITD^+^ patients showed persistent accumulation of mDC/pDC precursors and a decline in the levels of terminal mDCs who relapsed after 15 months

**Fig. 4:**
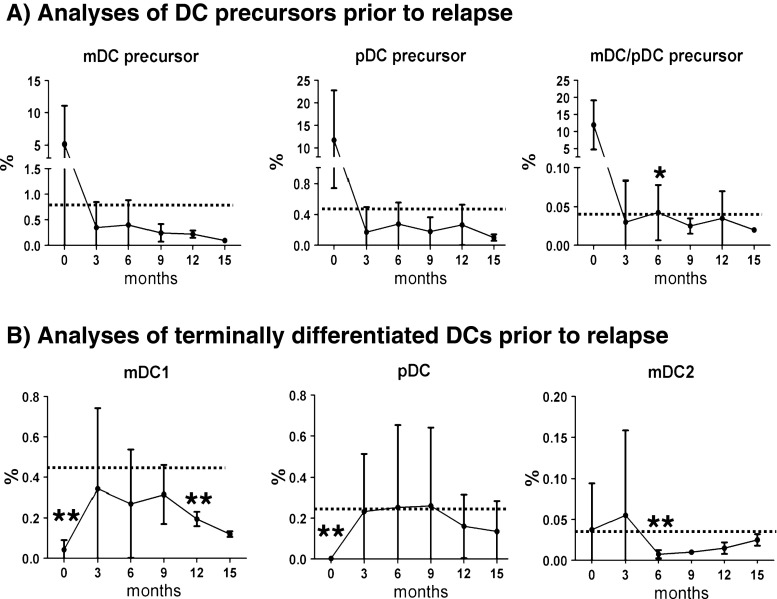
Kinetic analyses of DC patterns during CR of ITD^+^ AML patients (*n* = 4) showed persistent accumulation of mDC/pDC precursors and a decline in the levels of terminal mDC1 prior to relapse. **a** Frequencies of precursor DCs at 0, 3, 6, 9, 12, and 15 months. **b** Frequencies of terminal DCs at 0, 3, 6, 9, 12, and 15 months. *Single asterisk:* significantly higher than HD (*p* < 0.05). *Double asterisk:* significantly lower than HD (*p* < 0.05). [The *dotted line* is showing the average values for HD]

We chose four patients who experienced leukemia relapse within 2 years after FD (see Table 1, CR/REL patients 6, 7, 8, and 9) for performing kinetic analyses. PBMC samples were collected five times sequentially after FD (every 3 months and up for 15 months). The frequencies of precursor mDCs and pDCs normalized during the first 6 months of CR (although always lower than healthy controls), but then decreased continuously until 15 months (Fig. 4a). The mixed lineage mDC/pDC population drastically decreased during CR, but was nevertheless still detectable throughout the observation period (Fig. 4a). The frequencies of terminal mDC1s were always lower compared to HD controls, whereas terminal mDC2s returned to normal levels at 15 months (Fig. 4b). The frequencies of terminal pDCs did not reveal significant fluctuations during CR (Fig. 4b). Altogether, these results showed that for patients who relapsed, alterations in mDC frequency persisted throughout the remission period. Notably, both precursor and terminally differentiated mDC tended to disappear prior to relapse.

### IL-10 and stress cytokines secreted by CR/REL samples were observed as a later event, preceding relapse

**Fig. 5:**
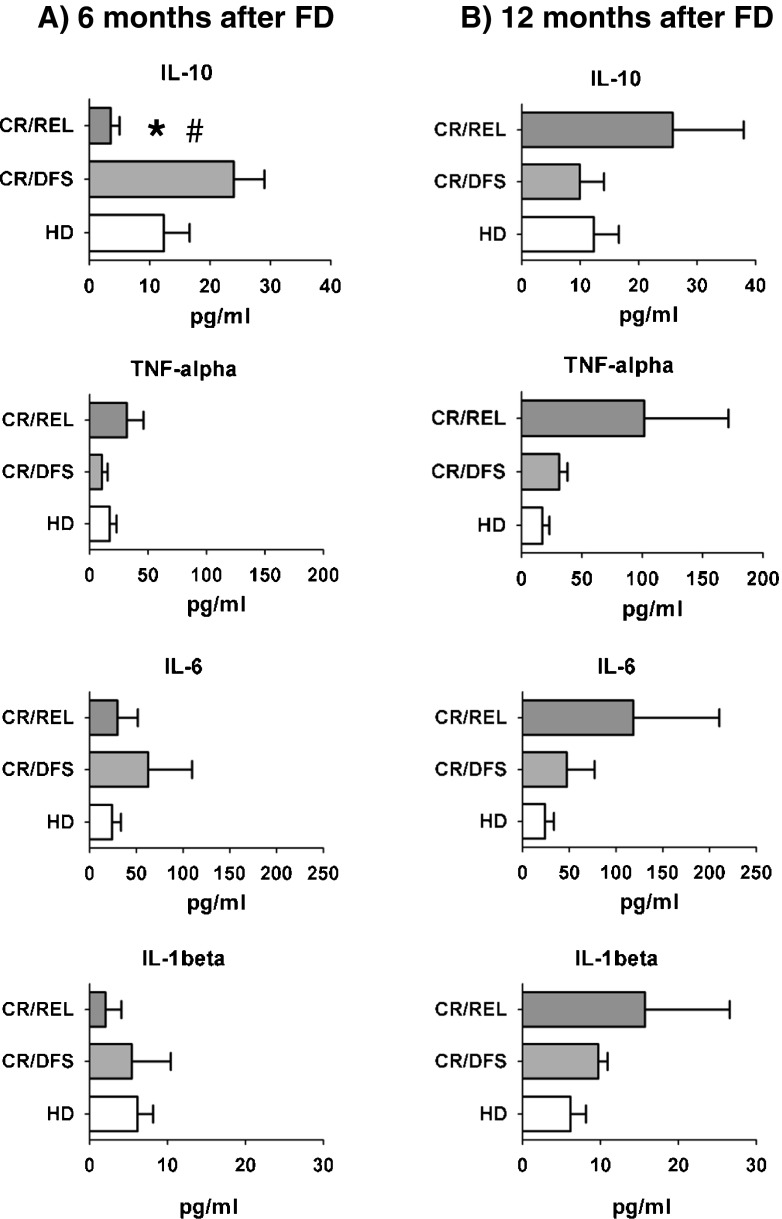
Secretion of stress cytokines by PBMC samples of CR/REL samples (*n* = 4) was observed as a later event (12 months after FD), yet several months prior to relapse. CR/DFS patients (*n* = 3) were not significantly different to healthy donors (HD, *n* = 4). **a** Cytokines secreted at 6 months after FD, comparing CR/REL, CR/DFS and HD. **b** Cytokines secreted at 12 months after FD, comparing CR/REL, CR/DFS, and HD. *Single asterisk:* significantly lower than CR/DFS (*p* < 0.05). *Number sign:* trend for lower secretion compared to HD (*p* = 0.0988). [Detection level for cytokines is 3.2 pg/ml]

We had observed that secretion of IL-10, TNF-α, IL-6, and IL-1β cytokines in FD samples of ITD^+^ nonresponder patients was higher than for patients who entered CR (Fig. 2), but we could not discern if the leukemic cells or possible bystander cells were producing the cytokines. IL-10 and stress cytokines have been linked with the occurrence of myeloid-derived suppressor cells (MDSCs), which can hinder DC development from myeloid precursor cells [27–29]. Since CR leukemic cells were not detectable in PBMC samples by morphology, we investigated if non-leukemic cells contained in the PBMC samples might lead to aberrant cytokine production. We used samples from the same four CR/REL patients described above for the cytokine secretion patterns at earlier (6 months, Fig. 5a) and later time points (12 months, Fig. 5b) after FD. As controls, we included samples obtained from three CR/DFS patients and four HD in the analyses. At 6 months after FD, IL-10 secretion by PBMCs of CR/REL samples was significantly lower than for samples from CR/DFS patients and HD, but the levels of the stress cytokines were comparable (Fig. 5a). Yet, at 12 months after FD, there was a trend for higher production of all stress cytokines in CR/REL samples in comparison with CR/DFS patients and HD samples (Fig. 5b). Nevertheless, results correlated with the lower frequencies of mDCs observed in CR/REL patients 12 months after FD (Fig. 4) suggesting that increased production of stress cytokines by cells contained in the PBMC samples was linked to decreased mDC differentiation. Since the PBMC samples at 12 months did not show detectable leukemic blasts, this finding underscored our hypothesis that other cells, such as myeloid precursor cells or myeloid-derived suppressor cells, could be implicated in the secretion of the cytokines.

### Soluble factors produced by the ITD^+^ FD samples can increase viability and stimulate monocytes to secrete IL-10 and stress cytokines

**Fig. 6:**
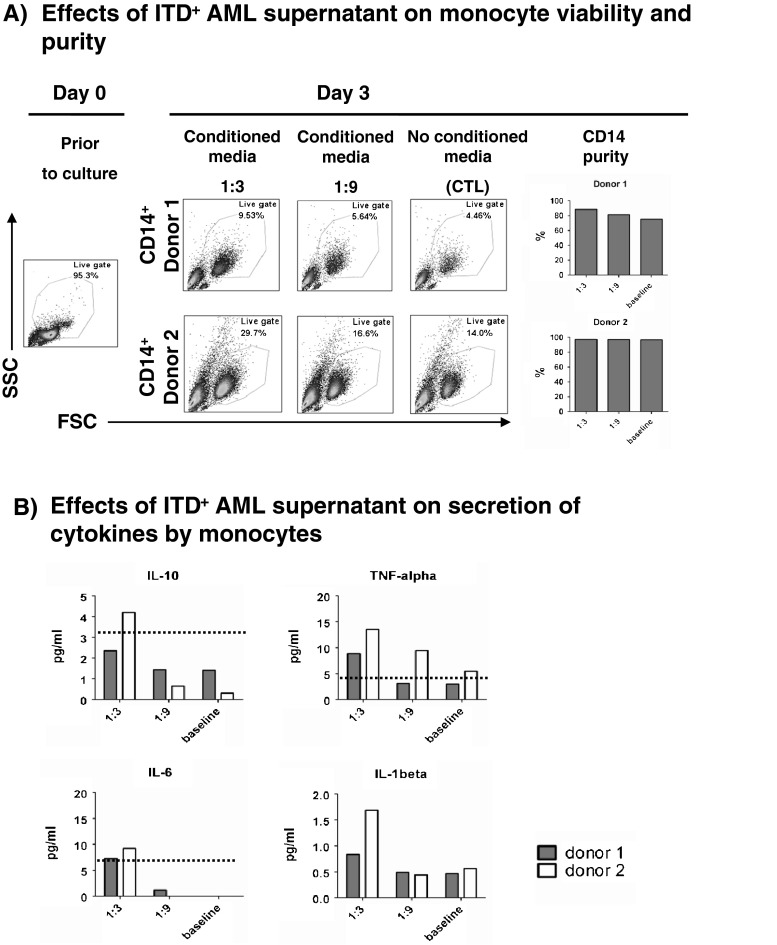
Soluble factors produced by ITD^+^ AML PBMCs (patient #6) can increase viability and stimulate monocytes to secrete stress cytokines. **a** ITD^+^ AML supernatant increases the viability of monocytes in vitro, as shown on day 3 of culture, and preserves the expression of CD14. **b** ITD^+^ AML supernatant stimulates monocytes to produce stress cytokines after 3 days of co-culture. [Detection level for cytokines is 3.2 pg/ml]

It was previously shown that cell supernatants obtained from leukemic cells inhibited DC differentiation ex vivo, promoted by recombinant cytokines [30]. We set up similar assays with cell culture supernatants obtained from an ITD^+^ FD sample and from a leukemic cell line. However, we did not observe inhibition of DC differentiation (data not shown). We thus evaluated the effects of leukemic cell supernatants on CD14^+^ monocytes, which are myeloid precursors of mDC. Cell supernatant collected from an ITD^+^ FD sample (obtained from CR/REL patient #6 (Table 1)) was used as conditioned media for the culture of CD14^+^ monocytes obtained from two healthy donors (enriched to 90–95 % purity with immunoconjugated magnetic beads). The experiments were performed using 1:3 and 1:9 leukemic supernatant dilutions in order to evaluate a possible dose-dependent effect. A mock baseline control was run in parallel. The monocytes were collected after 3 days of culture and analyzed for viability and purity, while the supernatants were analyzed for cytokine production. For the two independent experiments, FD leukemia supernatant at 1:3 dilution doubled the viability of monocytes compared to the 1:9 dilution or the mock control (Fig. 6a). Correlating with the increased monocyte viability, the leukemia supernatant (1:3 dilution) induced the secretion of IL-10, TNF-α, IL-6, and IL-1β in both experiments (Fig. 6b).

## Discussion

Deregulated dendropoiesis and DC dysfunction have been acknowledged in recent years to trigger immune evasion and tolerance induction against cancer [13, 31, 32]. Incomplete regeneration of DC populations in leukemia patients after stem cell transplantation has also been described in the literature as a negative prognostic factor [25, 26]. We have previously demonstrated that the presence of the ITD mutation in AML was correlated with an aberrant accumulation of cells with hallmarks of arrested dendritic cells [14]. We hypothesized that constitutive signaling through FLT3-ITD could arrest MRD leukemic dendritic cell precursors in an immature stage. In this study, we confirmed and extended our previous observation and hypothesis, as leukemic ITD^+^ DC did not show expression of molecules used routinely as markers for quantification of differentiated myeloid or plasmacytoid DC in peripheral blood (BDCA-1, BDCA-2, and BDCA-3).

Furthermore, we proposed to evaluate the patterns of dendritic cell lineages and of a subset of immunomodulatory cytokines (IL-10, TNF-α, IL-6, and IL-1β) during the course of ITD^+^ leukemia. We obtained samples from patients treated with conventional induction/consolidation chemotherapy, from the first diagnosis to eventual relapse.

**Fig. 7:**
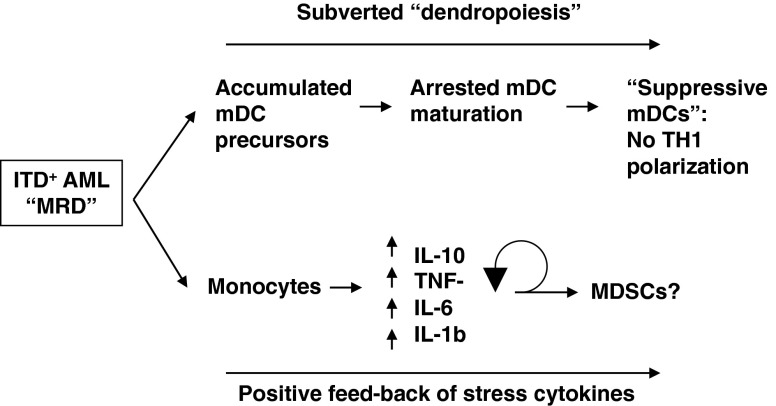
Proposed model of ITD^+^ AML inhibition of DC differentiation and activation of myeloid-derived suppressor cells

Our results indicated an incomplete regeneration of the myeloid dendritic cell compartment in ITD^+^ leukemia patients, which was aggravated prior to relapse (Suppl. Fig. 3). Notably, the decreased frequencies of mDCs in PBMC samples obtained prior to relapse were correlated with increased secretion of IL-10 and stress cytokines (TNF-α, IL-6, and IL-1β) in comparison to samples obtained from patients who did not relapse. The fact that leukemic cells could not be detected in these pre-relapse PBMC samples indicated that non-leukemic cells were involved with secretion of these cytokines. In order to experimentally test this, we demonstrated in a 3-day assay that cell-free supernatants obtained from an ITD^+^ diagnostic sample could activate monocytes obtained from healthy donors to secrete IL-10, TNF-α, IL-6, and IL-1β. Based on these observations, we hypothesize that suppression of dendropoiesis during remission of ITD^+^ leukemia is mediated by soluble factors (produced by minimal residual leukemia or by the leukemia microenvironment) that activate myeloid cells (such as monocytes) to produce IL-10, IL-6, TNF-α, and IL-1β. These cytokines in turn could deregulate the differentiation and maturation of myeloid DC (Fig. 7).

IL-6 was defined as a crucial factor for tumor survival and immune evasion, i.e., via induced secretion of IL-10 by regulatory T cells, which effectively suppresses adaptive immune responses towards tumor antigens [33] and induces proliferation of immature myeloid cells [34]. The combination of IL-6 and its inducers TNF-α and IL-1β [35] promotes tumor growth and survival in cancer patients through maintenance of chronic inflammation and induction of a tumor-supporting microenvironment [19, 36].

Prior to our work, supernatants of FD leukemia samples containing IL-6 and IL-10, TNF-α, and IL-1β were shown to trigger a block in DC differentiation in vitro [30]. These results corroborate in part with ours, as we observed high levels of these cytokines only in FD samples of patients who did not enter remission. In our experimental setting, the FD leukemia supernatants did not block ex vivo differentiation of conventional myeloid DC (grown in the presence of GM-CSF and IL-4, results not shown). Nevertheless, we were able to show an effect of the FD leukemia supernatants on monocyte viability as a feedback loop for production of these same cytokines. It is important to note that IL-6 and IL-10, TNF-α, and IL-1β have been implied in the generation of the MDSCs, which are commonly found in cancer patients [27–29, 37, 38]. MDSCs are a heterogeneous group of immature myeloid cells arrested in an early differentiation step towards becoming a DC and defined by their immunosuppressive capacities [38–40], which include direct inhibition of tumor-reactive T cells [27, 41]. Interestingly, the ITD mutation itself was shown to be implied in certain regulatory mechanisms, i.e., by induction of increased reactive oxygen species production [42], which is regulating a major mechanism of immune suppression in cancer as induced by MDSCs [37].

Noteworthy, a previous study evaluated serum levels of TNF-α, IL-6, and IL-10, in diagnostic samples obtained from patients with AML or high-risk myelodysplastic syndromes. Lower TNF-α levels were found to significantly correlate with better performance status, whereas the other cytokines were not found to be predictive of clinical outcomes [43]. Blockage of TNF-α is already used as standard treatment in various autoimmune diseases [35], whereas clinical trials to evaluate blockage of IL-6 and IL-1β in inflammatory diseases and cancer are ongoing [35, 44–46]. Thus, evaluation of novel immunomodulatory approaches like these may be also warranted for improving survival of ITD^+^ AML patients.

In conclusion, lack of mature mDCs in ITD^+^ FD patient samples in combination with the incomplete regeneration of mDCs during remission and higher secretion of IL-10 and stress cytokines (TNF-α, IL-6, and IL-1β) in patients who relapsed strengthen the hypothesis of immunomodulatory changes during the course of ITD^+^ AML relapse. These events are likely to be linked with a “subverted dendropoiesis” and induction of MDSC in these leukemia patients (Fig. 7). Thus, clinical monitoring of PBMC samples of ITD^+^ patients during remission in order to enumerate the DC profile and to detect the secretion of IL-10 and stress cytokine levels as well as the occurrence of MDSCs might serve as immunologic biomarkers to provide additional approaches for disease staging.

## Electronic supplementary material

Suppl. Fig. 1 FACS analyses validation with cryopreserved samples and sample collection at different stages of disease. **a** Validation of immunophenotypic assay to determine DC frequencies in cryopreserved PBMCs from healthy donors (*n* = 9). **b** Schedule and clinical definitions of PBMC samples collected from AML patients. (PPT 174 kb)

Suppl. Fig. 2 Example of aberrant DC distribution in FD samples obtained from a representative ITD+ AML patient in comparison with a healthy donor. **a** Accumulation of precursor DCs (mixed lineage mDC/pDC). **b** Lack of terminal DCs. (PPT 352 kb)

Suppl. Fig. 3 Alteration in DC frequencies and production of stress cytokines by PBMCs are correlated with the outcome of ITD+ AML patients [Arrows indicating higher or lower frequencies/secretion]. (PPT 91 kb)

Suppl. Table 1 Patient characteristics for the ITD+ and ITD– AML patient cohorts. (DOC 28 kb)
