# Supplementary material for: Monitoring dendritic cell and cytokine biomarkers during remission prior to relapse in patients with FLT3-ITD acute myeloid leukemia
Source: Ann Hematol. 2013 Apr 25;92(8):1079–90. doi: 10.1007/s00277-013-1744-y (PMC3701796; doi:10.1007/s00277-013-1744-y)
Supplement: Supplementary file 1 — FACS analyses validation with cryopreserved samples and sample collection at different stages of disease. a Validation of immunophenotypic assay to determine DC frequencies in cryopreserved PBMCs from healthy donors (n = 9). b Schedule and clinical definitions of PBMC samples collected from AML patients. (PPT 174 kb) [file 277_2013_1744_MOESM1_ESM.ppt]

## Slide 1
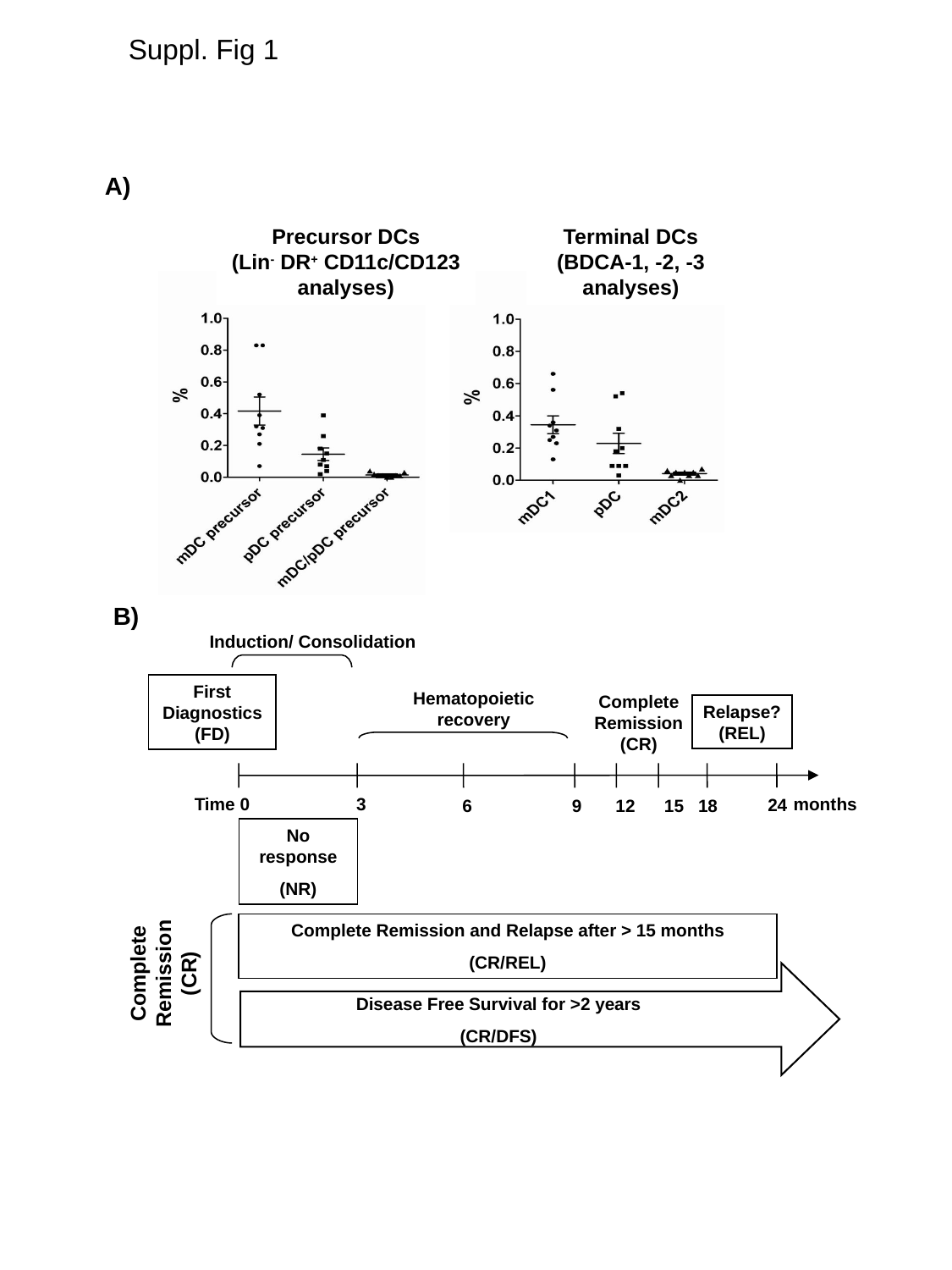

Suppl. Fig 1
A)
Precursor DCs
(Lin- DR+ CD11c/CD123 analyses)
Terminal DCs
(BDCA-1, -2, -3
analyses)
B)
Induction/ Consolidation
First Diagnostics
(FD)
Hematopoietic recovery
Complete
Remission
(CR)
Relapse?
(REL)
months
Time 0
3
24
6
9 12 15
18
No response
(NR)
Complete Remission and Relapse after > 15 months
(CR/REL)
Complete Remission (CR)
Disease Free Survival for >2 years
(CR/DFS)
