# Supplementary material for: Monitoring dendritic cell and cytokine biomarkers during remission prior to relapse in patients with FLT3-ITD acute myeloid leukemia
Source: Ann Hematol. 2013 Apr 25;92(8):1079–90. doi: 10.1007/s00277-013-1744-y (PMC3701796; doi:10.1007/s00277-013-1744-y)
Supplement: Supplementary file 2 — Example of aberrant DC distribution in FD samples obtained from a representative ITD+ AML patient in comparison with a healthy donor. a Accumulation of precursor DCs (mixed lineage mDC/pDC). b Lack of terminal DCs. (PPT 352 kb) [file 277_2013_1744_MOESM2_ESM.ppt]

## Slide 1
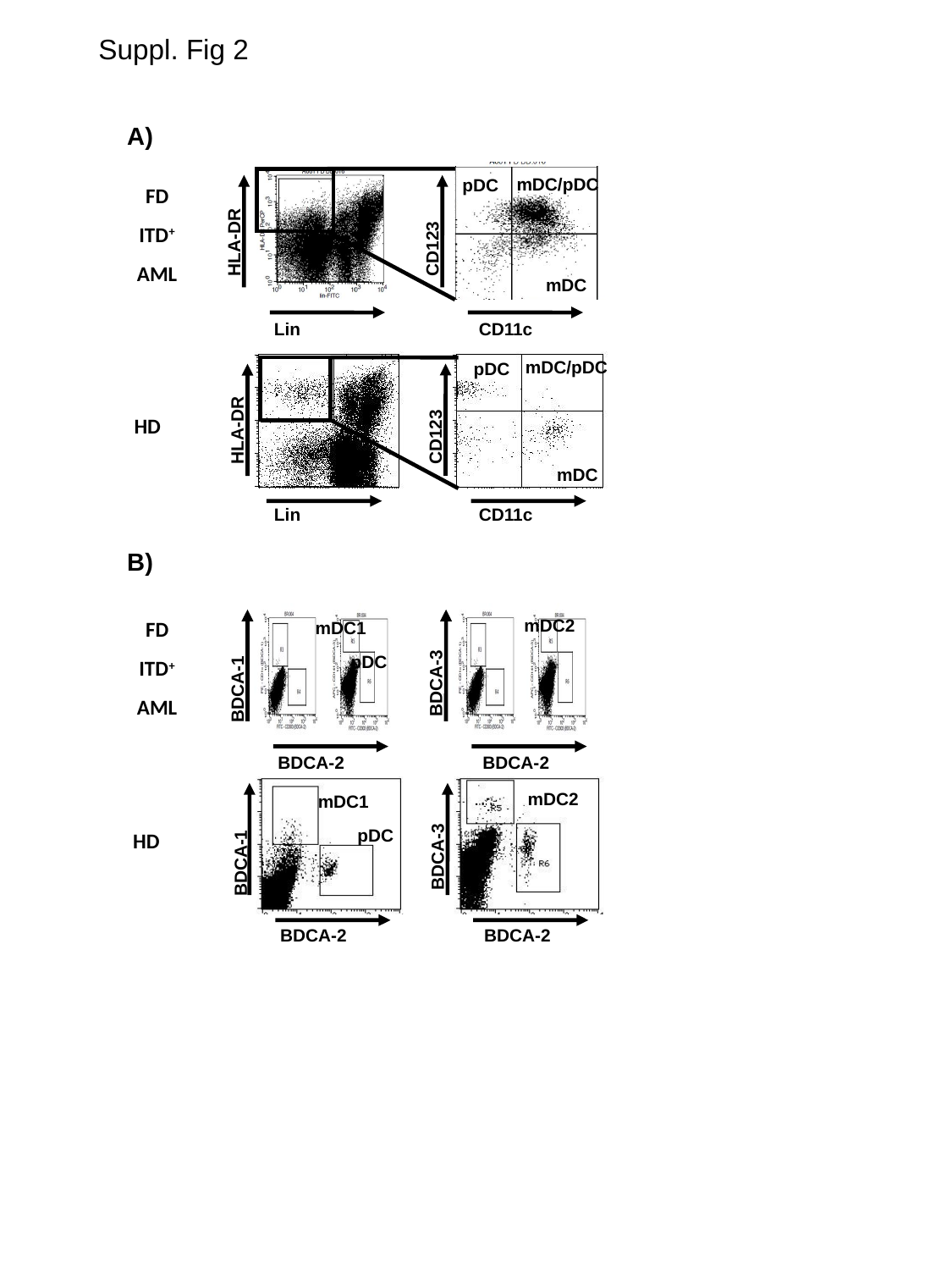

Suppl. Fig 2
A)
mDC/pDC
pDC
FD
ITD+
AML
HLA-DR
CD123
mDC
Lin
CD11c
mDC/pDC
pDC
HLA-DR
CD123
HD
mDC
Lin
CD11c
B)
mDC2
FD
ITD+
AML
mDC1
BDCA-3
pDC
BDCA-1
BDCA-2
BDCA-2
mDC2
mDC1
BDCA-3
pDC
HD
BDCA-1
BDCA-2
BDCA-2
