# Supplementary material for: Monitoring dendritic cell and cytokine biomarkers during remission prior to relapse in patients with FLT3-ITD acute myeloid leukemia
Source: Ann Hematol. 2013 Apr 25;92(8):1079–90. doi: 10.1007/s00277-013-1744-y (PMC3701796; doi:10.1007/s00277-013-1744-y)
Supplement: Supplementary file 3 — Alteration in DC frequencies and production of stress cytokines by PBMCs are correlated with the outcome of ITD+ AML patients [Arrows indicating higher or lower frequencies/secretion]. (PPT 91 kb) [file 277_2013_1744_MOESM3_ESM.ppt]

## Slide 1
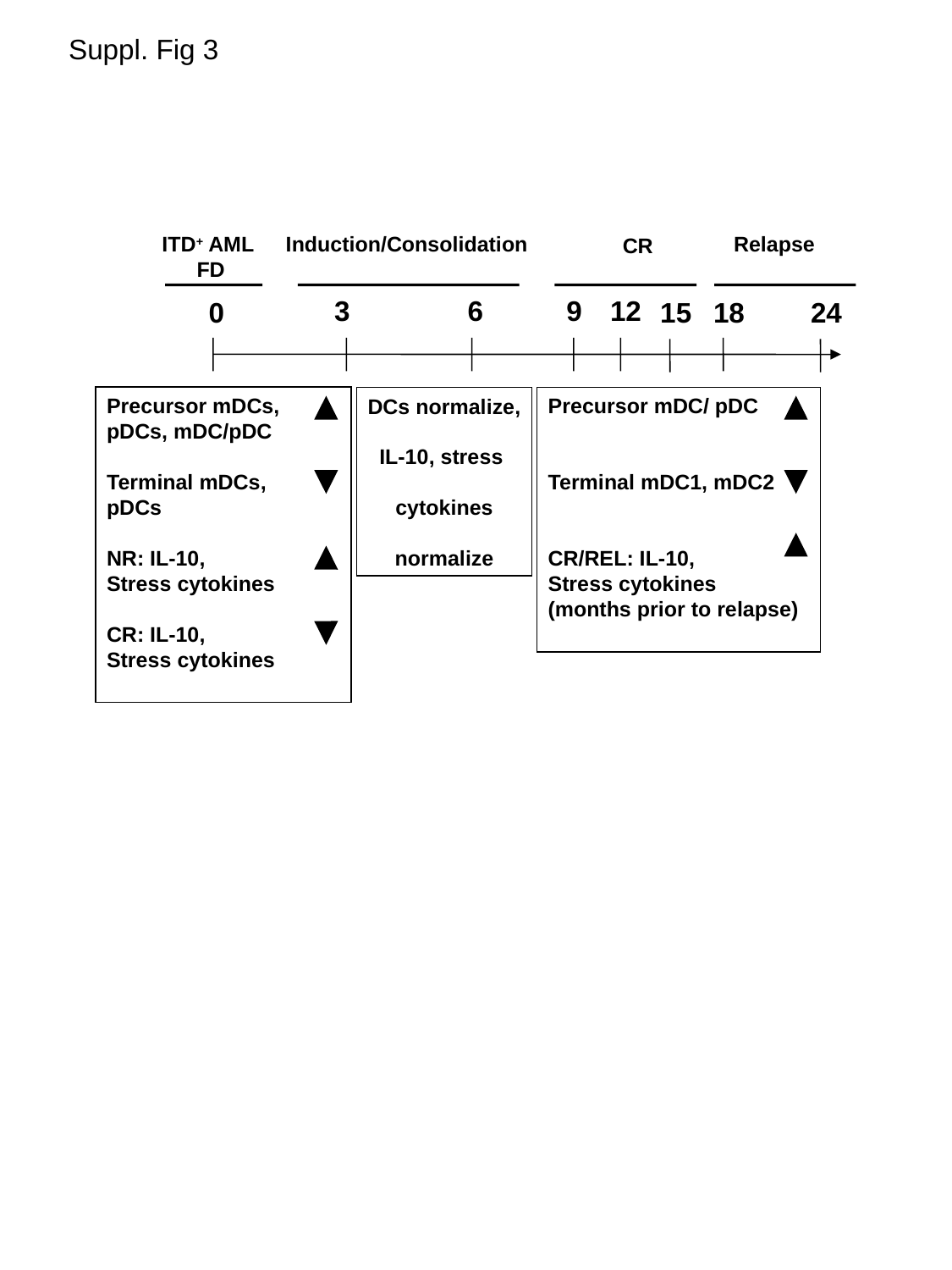

Suppl. Fig 3
Relapse
ITD+ AML
FD
Induction/Consolidation
CR
3
6
9
12
0
15
18
24
Precursor mDCs,
pDCs, mDC/pDC
Terminal mDCs,
pDCs
NR: IL-10,
Stress cytokines
CR: IL-10,
Stress cytokines
DCs normalize,
IL-10, stress
cytokines
normalize
Precursor mDC/ pDC
Terminal mDC1, mDC2
CR/REL: IL-10,
Stress cytokines
(months prior to relapse)
