# Supplementary material for: Monitoring dendritic cell and cytokine biomarkers during remission prior to relapse in patients with FLT3-ITD acute myeloid leukemia
Source: Ann Hematol. 2013 Apr 25;92(8):1079–90. doi: 10.1007/s00277-013-1744-y (PMC3701796; doi:10.1007/s00277-013-1744-y)
Supplement: Supplementary file 4 — Patient characteristics for the ITD+ and ITD– AML patient cohorts. (DOC 28 kb) [file 277_2013_1744_MOESM4_ESM.doc]

**Suppl. Table 1** Patient characteristics for the ITD^+^ and ITD^-^ AML patient cohorts.

ITD+ ITD-

(n=26) (n=28)

Gender

Male (%) 13 (50) 18 (64)

Female (%) 13 (50) 10 (36)

Median age (range)

54.4 (18-83) 56.4 (31-82)

Median WBC at FD (range)

69.1 (0.4-119.2) 63.1 (1.4-146.3)

NPM1 Mutation (%)

15 (58) 11 (40)

FAB subtype (%)

M1 1 (4) 2 (7)

M2 2 (8) 0 (0)

M3 1 (4) 2 (7)

M4 8 (32) 7 (25)

M5 6 (24) 4 (14)

M6 1 (4) 0 (0)

Secondary AML 1 (4) 3 (11)

Not available 5 (20) 10 (36)

Cytogenetics (%)

Normal 17 (65) 14 (50)

Complex 3 (12) 6 (22)

APL 1 (4) 2 (7)

Inv(16) 0 (0) 2 (7)

Not available 5 (19) 4 (14)

Median DFS in months

24.7 39.3

Disease progression (%)

No CR 4 (16) 0 (0)

REL 8 (32) 12 (43)

SCT 11 (42) 7 (25)

Outcome (%)

Alive 14 (54) 17 (61)

Dead 9 (35) 2 (7)

Not available 3 (11) 9 (32)

Legend: WBC: white blood cells x1000/μl; FD: first diagnosis; NPM1 Mutation: Exon 12 Nucleophosmin Mutation; FAB: French American British Classification; DFS: disease-free survival in months (follow-up until June 2012); CR: complete remission; REL: relapse; SCT: stem cell transplantation;
